# Naringin and Hesperidin Counteract Diclofenac-Induced Hepatotoxicity in Male Wistar Rats *via* Their Antioxidant, Anti-Inflammatory, and Antiapoptotic Activities

**DOI:** 10.1155/2021/9990091

**Published:** 2021-08-11

**Authors:** Rasha A. Hassan, Walaa G. Hozayen, Haidy T. Abo Sree, Hessah M. Al-Muzafar, Kamal A. Amin, Osama M. Ahmed

**Affiliations:** ^1^Department of Biochemistry, Faculty of Science, Beni-Suef University, P.O. Box 62521, Beni-Suef, Egypt; ^2^Faculty of Oral and Dental Medicine, Nahda University, Beni-Suef, Egypt; ^3^Chemistry Department, College of Science, Imam Abdulrahman Bin Faisal University, P.O. Box 1982, Dammam 31441, Saudi Arabia; ^4^Basic & Applied Scientific Research Center, Imam Abdulrahman Bin Faisal University, P.O. Box 1982, Dammam 31441, Saudi Arabia; ^5^Physiology Division, Department of Zoology, Faculty of Science, Beni-Suef University, P.O. Box 62521, Beni-Suef, Egypt

## Abstract

This study is aimed at evaluating the preventive effect and at suggesting the mode of actions of naringin and hesperidin and their combination in diclofenac-induced hepatotoxicity. Male Wistar rats, intraperitoneally injected with diclofenac sodium (3 mg/kg b.wt/day), were orally treated with naringin (20 mg/kg b.wt/day) and hesperidin (20 mg/kg b.wt/day) and their combination for 4 weeks. The administrations of naringin and hesperidin to diclofenac-injected rats led to a significant decrease in the elevated serum ALT, AST, LDH, ALP, GGT, total bilirubin, TNF-*α*, and IL-17 levels as well as liver lipid peroxidation and liver p53 and caspase-3 mRNA expressions. In contrast, serum IL-4 level, liver GSH content, and liver GPx and SOD activities increased. In association, diclofenac-induced deleterious histological alterations including hydropic degeneration, cytoplasmic vacuolization, apoptosis, and focal hepatic necrosis of hepatocytes associated with inflammatory cells' infiltration were remarkably improved by treatments with naringin and hesperidin. In conclusion, naringin, hesperidin, and their combination, which was the most potent, counteract diclofenac-induced liver injury *via* antioxidant, anti-inflammatory, and antiapoptotic actions. Thus, this study recommends the use of naringin and hesperidin or their combination to resolve the side effects of drugs like diclofenac on the liver.

## 1. Introduction

Nonsteroidal anti-inflammatory drugs )NSAIDs) have various side effects [1]. Diclofenac, as one of this category of drugs, is a phenylacetic acid derivative that has long been used as analgesic and an anti-inflammatory drug to treat certain conditions [2]. In its mechanism of action, diclofenac inhibits cyclooxygenase- (COX-) 2 enzymes with greater potency than it acts on COX-1 [3]. Although diclofenac has potent therapeutic effects, its sustained use is associated with serious dose-dependent adverse effects [3]. Increase in the oxidative stress and suppression of antioxidant defense system were reported by various publications that they were implicated in the induction of toxicity and side effects of the drug [4, 5]. The use of antioxidant such as citrus flavonoid in combination with the diclofenac may counteract the diclofenac-induced oxidative stress and thereby may prevent drug side effects and toxicity.

Naringin responsible for the sour flavor and bitter taste of the fruits of *Citrus species* have broad-spectrum pharmacological and therapeutic properties as lipid lowering, anti-inflammatory, free radical-scavenging, antioxidative, antihyperlipidemic, antiapoptotic, and antiatherogenic properties [6, 7]. Hesperidin is another flavonoid isolated from *Citrus species* that had several biological properties, particularly antioxidant and anti-inflammatory [8]. It has been reported that hesperidin improved the cytochrome-induced liver tissue injury observed by histopathological examination [9]. It was also revealed that hesperidin was effective in suppressing inflammation and allergic symptoms and in the treatment of allergic rhinitis [10].

Therefore, this study was conducted to assess the possible preventive effects of naringin and hesperidin on diclofenac-induced liver toxicity and to explore the roles of modulation of inflammation, oxidative stress, and apoptosis in the preventive action.

## 2. Materials and Methods

### 2.1. Chemicals and Kits

Diclofenac sodium was obtained from El-Nasr Pharmaceutical Chemical Company, Egypt. Naringin and hesperidin were produced by Sigma-Aldrich Company (St. Louis, MO, USA). All other used chemicals are of analytical grade and were obtained from local chemical companies. Alanine transaminase (ALT) and aspartate transaminase (AST) kits were obtained from Spinreact, Ctra Sta Coloma, 7. 17176 St., Esteve de Bas, Girona, Spain. Lactate dehydrogenase (LDH) and *γ*-glutamyltransferase (GGT) was obtained from Spectrum, Egyptian Company for Biotechnology, Obour City Industrial Area, Cairo, Egypt. Alkaline phosphatase (ALP) kit was obtained from Biodiagnostic, 29 Tahrir St., Dokki, Giza, Egypt. Albumin and bilirubin kits were obtained from Diamond, 24 El Montazah St., Heliopolis, Cairo, Egypt.

### 2.2. Experimental Animals

Adult male rats of Wistar strain weighing 100-130 g (9-11 weeks) were used as experimental animals in the present work. They were obtained from Animal House of Ophthalmology Research Institute, Giza, Egypt. The animals were housed in good aerated cages at 12 hours daily of light-dark cycles and temperature between 20 and 25°C. Rats were supplemented daily with standard pelleted diet and water *ad libitum*. The animals were maintained for two weeks under observation before starting the experiment for adaptation and to exclude any intercurrent infection. All animal methodologies are in accordance with the guidelines and instructions of the Experimental Animals Ethics Committee of the Faculty of Science for Care and Use of the Animals, Beni Suef University, Egypt. The ethical approval number is BSU/FS/2015/15. All efforts were done to reduce the suffering and distress of animals.

### 2.3. Animal Grouping

**Figure 1:**
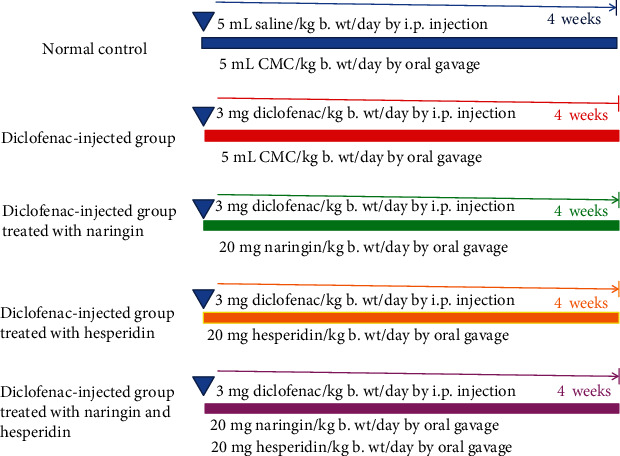
Experimental design and animal grouping. i.p.: intraperitoneal.

Thirty male Wistar rats were used in this experiment and were allocated into five groups (Figure 1). The rats of group 1 (normal) received the equivalent volume of saline (the vehicle in which diclofenac was dissolved) by intraperitoneal injection and also given the equivalent volume of 1% carboxymethyl cellulose (CMC; the vehicle in which naringin and hesperidin was dissolved) by oral gavage daily for 4 weeks. Group 2 (diclofenac-injected group) was injected with diclofenac (dissolved in saline) by intraperitoneal administration at a dose of 3 mg/kilogram body weight (kg b. wt)/day [11] and was also given the equivalent volume of 1% CMC by oral gavage daily for 4 weeks. Group 3 (diclofenac-injected group treated with naringin) was given diclofenac as in group 2 and was orally treated with naringin (dissolved in 1% CMC) at a dose level of 20 mg/kg b. wt/day by oral gavage [12] for 4 weeks. Group 4 (diclofenac-injected group treated with hesperidin) was given diclofenac as in group 2 and was orally treated with hesperidin (dissolved in 1% CMC) at a dose of 20 mg/kg b. wt/day [13] for 4 weeks. Group 5 (diclofenac-injected group treated with naringin and hesperidin) was supplemented with diclofenac as in group 2 and was orally treated with naringin and hesperidin (dissolved in 1% CMC) at a dose of 20 mg/kg b. wt/day for 4 weeks.

### 2.4. Blood and Liver Sampling

At the end of the experiment, rats deprived of food and blood samples were collected from jugular vein. The blood samples were left to coagulate at the room temperature and then centrifuged at 3000 rpm for 15 minutes. The serum of each blood sample was fractioned into three vials that were kept at -30°C until used for biochemical analysis.

After taking blood samples from jugular vein, the rats were euthanized and dissected. The liver from each rat was rapidly excised and washed in sterile saline (0.9% NaCl). Pieces of the liver were fixed in 10% neutral buffered formalin for histological investigation. Other parts were kept in deep freezer at -30°C pending homogenization in sterile isotonic saline (0.9% NaCl) for determination of oxidative stress and antioxidant defense markers. Third parts of the liver were kept at -30°C in sterile Eppendorf tubes for reverse transcriptase-polymerase chain reaction (RT-PCR) analysis.

### 2.5. Detection of Liver Function Parameters in Serum

ALT and AST activities were estimated using the nicotinamide adenine dinucleotide hydrogen (NADH) oxidation method according to methods of Murray [14] and Murray [15], respectively, using reagent kits purchased from Spinreact, Spain. In brief, ALT catalyzes the conversion of alanine to pyruvate, which is reduced to lactate by LDH, and NADH is oxidized to nicotinamide adenine dinucleotide (NAD). The rate of decrease in concentration of NADH, measured photometrically at 340 nm, is proportional to the catalytic concentration of ALT present in the serum sample. On the other hand, AST catalyzes the conversion of aspartate to oxaloacetate, which is reduced to malate by malate dehydrogenase (MDH), and NADH is oxidized to NAD. The rate of decrease in concentration of NADH, measured photometrically at 340 nm, is proportional to the catalytic concentration of AST present in the serum sample. Serum LDH activity was detected according to method of Van der Heiden et al. [16]. In this method, LDH catalyzes the reaction between pyruvate and NADH to produce NAD^+^ and L-lactate. The rate of decrease in concentration of NADH, measured photometrically at 340 nm, is proportional to the catalytic concentration of LDH present in the sample. Serum ALP activity was determined according to method of Belfield and Goldberg [17] using kits obtained from Biodiagnostic, Egypt. By adding serum sample to phenyl phosphate (5 mmol/L) at pH 10, ALP leads to formation of phenol and phosphate. The liberated phenol is measured colorimetrically at 510 nm in the presence of 4-aminophenazone and potassium ferricyanide. GGT activity was determined according to method of Szasz and Persijn [18] using kits obtained from Spectrum Diagnostic, Egyptian Company for Biotechnology, Egypt. GGT transfers the *γ*-glutamyl group from the substrate L-*γ*-glutamyl-p-nitroanilide, liberating the chromogen p-nitroanilide (pNA) which is measured at 418 nm and its formation is proportional to the GGT activity. Serum albumin level was determined according to the method of Doumas et al. [19] using reagent kits purchased from Diamond, Egypt. The method in brief was performed by adding 10 *μ*L serum to 2 mL bromocresol green (0.12 mmol/L) in citrate buffer pH 3.8. The albumin/bromocresol green complex, formed by the reaction, was measured at 630 nm. Serum bilirubin level was assayed according to methods of Malloy and Evelyn [20] using reagent kits purchased from Diamond, Egypt. This method for bilirubin estimation is based on van den Bergh reaction. In this reaction, bilirubin reacts with diazotized sulfanilic acid to produce azobilirubin which is purple in color which was measured photometrically at 578 nm. Intensity of color is directly proportional to the amount of bilirubin in the serum.

### 2.6. Evaluation of Liver Oxidative Stress and Antioxidant Defense System Parameters

Liver lipid peroxidation (LPO) was determined according to method of Preuss et al. [21]. In brief, the protein was precipitated by adding 0.15 mL 76% trichloroacetic acid (TCA) to 1 mL liver homogenate. Then, 0.35 mL of thiobarbituric acid (TBA) was added, as a color-developing agent, to the separated supernatant. The developed faint pink color was measured at 532 nm after incubation in water bath at 80°C for 30 minutes. MDA (malondialdehyde or 1,1,3,3-tetramethoxypropane) was used as standard. Reduced glutathione (GSH) content in the liver was determined by adding 0.5 mL 5,5′-dithiobis (2-nitrobenzoic acid), Ellman's reagent (as a color-developing agent), and phosphate buffer solution (pH 7) to homogenate supernatant after protein precipitation based on the procedure of Beutler et al. [22]. The developed yellow colors in samples and GSH standard were measured at 412 nm against blank. Liver GPx activity was measured according to the procedure of Matkovics et al. [23] with some modifications. This method is based on the detection of the GSH that is converted to oxidized glutathione (GSSG) by the enzyme through detection of the residual GSH and subtracting it from the total. Briefly, 50 *μ*L homogenate supernatant was added to a Wasserman tube containing 350 *μ*L Tris buffer (pH 7.6), 50 *μ*L GSH solution (2 mM), and 50 *μ*L H_2_O_2_ (3.38 mM). Then, after 10 minutes of incubation, the residual GSH content was measured by the previously described method for GSH determination at 430 nm. Standard test was prepared by adding 50 *μ*L distilled water instead of 50 *μ*L sample and blank test was prepared by adding 100 *μ*L distilled water instead of 50 *μ*L sample and 50 *μ*L GSH solution. After detection of residual GSH content in the sample, the GSH converted to oxidized form (GSSG) and the enzyme activity can be calculated. Liver superoxide dismutase (SOD) activity was detected according to the method of Marklund and Marklund [24]. The reaction is based on the inhibition of autooxidation of pyrogallol by SOD. The process is dependent on the presence of superoxide ions. The amount of the enzyme that causes a 50% inhibition in the extinction changes in 1 minute compared to the control is regarded as one unit of the enzyme.

### 2.7. Determination of Serum TNF-*α*, IL-17, and IL-4 Levels

Serum TNF-*α*, IL-17, and IL-4 levels were estimated according to specific enzyme-linked immunosorbent assay (ELISA) kits according to the manufacturer's instructions. Rat TNF-alpha ELISA kit (Catalog No.: MBS355371), IL-17 ELISA kit (Catalog No.: MBS5203506), and IL-4 ELISA kit (Catalog No.: MBS494192) were obtained from MyBioSource, Inc., San Diego, CA, USA. These ELISA kits use sandwich ELISA. In brief, the micro ELISA plates provided in these kits have been precoated with an antibody specific to the concerned cytokine. Standards or samples are added to the appropriate micro ELISA plate wells and combined with the specific antibodies. Then, a biotinylated detection antibody specific for the concerned cytokine and avidin-horseradish peroxidase (HRP) conjugate is added to each microplate well successively and incubated. Free components are washed away. The substrate solution is added to each well. Only those wells that contain cytokine, biotinylated detection antibody, and avidin-HRP conjugate will appear blue in color. The enzyme-substrate reaction is terminated by the addition of a sulphuric acid solution and the color turns yellow. The optical density (OD) is measured spectrophotometrically at a wavelength of 450 nm. The OD value is proportional to the concentration of the tested cytokine and its concentration in the samples can be determined by comparing the OD of the samples to the standard curve.

### 2.8. Detection of mRNA Expression of p53

#### 2.8.1. RNA Isolation

Total RNA was isolated from the liver tissue according to the method of Chomzynski and Sacchi [25] and Boom et al. [26] using Thermo Scientific GeneJET RNA purification kit obtained from Thermo Fisher Scientific Inc., Rochester, New York, USA. Briefly, liver samples are lysed and homogenized in lysis buffer, which contains guanidine thiocyanate as a chaotropic salt and *β*-mercaptoethanol capable of protecting RNA from endogenous RNases. The lysate is then mixed with ethanol and loaded on a purification column. The chaotropic salt and ethanol cause RNA to bind to the silica membrane while the lysate is spun through the column. Subsequently, impurities are effectively removed from the membrane by washing the column with wash buffers. Pure RNA is then eluted under low ionic strength conditions with nuclease-free water. The levels of isolated RNA were determined and quantified using ultraviolet (UV) spectrophotometer and taking the absorbance at optical densities (OD) at 260 nm and 280 nm. RNA was quantified and qualified based on Sambrook and Russell [27] formula (RNA (*μ*g/*μ*L) = OD at 260 nm × dilution × 40 *μ*g/mL/1000). For each extracted RNA sample, the ratio between OD at 260 nm and OD at 280 nm and the ratio ranged between 1.7 and 2.0 ensure high purity of extracted RNA.

#### 2.8.2. RT-PCR Assay

RT-PCR assay was applied using Thermo Scientific Verso 1-Step RT-PCR Reddy Mix Kit (Thermo Fisher Scientific Inc., Rochester, New York, USA) for detection of the mRNA expression of liver p53. In brief, in one step reaction, RNA was reverse transcribed into cDNA and the produced cDNA was amplified by Techne 32 Thermocycler. The reaction mixture consists of Veso Enzyme Mix (1 *μ*L), 2X One-Step PCR ReadyMix (25 *μ*L), RT enhancer (25 *μ*L), forward primer (10 *μ*M; 1 *μ*L), reverse primer (10 *μ*M; 1 *μ*L), template RNA (1 ng; 1.5 *μ*L), and water nuclease free to complete total volume to 50 *μ*L. The thermal cycling program includes cDNA synthesis (temp. 50°C, 15 minutes, 1 cycle), Verso inactivation (temp. 95°C, 2 minutes, 1 cycle), denaturation (temp. 95°C, 20 seconds), annealing (temp. 55°C, 30 seconds), extension (temp. 72°C, 1 minute), and final extension (temp. 72°C, 5 minutes, 1 cycle). The number of cycles of denaturation followed by annealing and extension is 35 cycles. After reverse transcription and amplification, 10 *μ*L of PCR products was analyzed on a 1.5% agarose gel stained with ethidium bromide in 1× Tris Borate EDTA buffer (TBE) pH 8.3–8.5. The electrophoretic picture was visualized and analyzed by gel documentation system (GelDocu Advanced), and values were normalized to the quantity of *β*-actin. The primer pair sequences p53 were 5'd CAGCGTGATGATGGTAAGGA 3' (forward) and 5'd GCGTTGCTCTGATGGTGA 3' (reverse) according to Asiri [28].

### 2.9. Histopathological Investigations

After sacrifice and dissection, the liver from each rat was rapidly excised and then perfused in saline solution. Pieces from the liver of each rat (3 mm^3^) were taken and fixed in 10% neutral buffered formalin for twenty-four hours. The fixed livers were sent to Pathology Department, Faculty of Veterinary Medicine, Beni Suef University, for further processing, blocking in wax, sectioning, and staining with haematoxylin and eosin (H&E) [29]. The stained liver sections were examined for detection of histopathological score. In three randomly selected fields of each section (×100), lesions or injuries were graded as absent (0), mild (I), moderate (II), and severe (III) for changes, 0%, less than 30%, 30-50%, and more than 50%, respectively [30]. The graded lesions included hydropic and vacuolar degeneration, inflammatory cell infiltration, necrosis, apoptosis, Kupffer cell proliferation, fatty changes and steatosis, congested blood vessels and sinusoids, and hyperplasia of epithelial lining of the bile duct.

### 2.10. Immunohistochemical Detection of Caspase-3

The paraffin-embedded liver samples were transferred to the Department of Pathology, National Cancer Institute, for sectioning into 5 *μ*m thick sections that were mounted on positive-charged slides (Fisher Scientific, Pittsburgh, PA). Caspase-3 reactivity was processed according to the methods of Galaly et al. [31] and Ahmed and Ahmed [32]. In brief, after antigen retrieval, diluted primary antibody for caspase-3 (Santa Cruz Biotechnology, Santa Cruz, CA, USA) was incubated with liver sections for 1 hour. Diluted biotinylated secondary antibody (DakoCytomation Kit) was added, and incubation was done for 15 minutes at 37°C. Thereafter, horseradish peroxidase conjugated with streptavidin (DakoCytomation Kit) was applied for further 15 minutes incubation. Bound antibody complex was visualized by the reaction of 3,3′-diaminobenzidine (DAB) substrate and counter staining with haematoxylin. All liver sections were incubated under the same conditions with the same dilutions of antibodies and at the same period, so the immunostaining was comparable among the different study groups. For each preparation, a negative control was performed (a slide without primary antibody). The sections were visualized under a light microscope, and the extent of cell immunopositivity was assessed. Images of sections of the liver were captured using a digital camera (Leica, DM2500M Leica, Wetzlar, Germany). Examination and analysis of labeling were performed using free software version ImageJ (1.51d) [33]. The ImageJ software was used to measure the integrated intensities (in pixels) of positive reaction of caspase-3.

### 2.11. Statistical Analysis

Results were represented in tables and graphs as the mean ± standard error (SE). The data were analyzed by LSD analysis to discern the main effects and to compare various groups with each other using PC-STAT, University of Georgia (USA) [34]. Values of *p* > 0.05 were considered statistically nonsignificantly different, while values of *p* < 0.05 were significantly and highly significantly different.

## 3. Results

### 3.1. Effect on Serum Parameters Related to Liver Function

The daily intraperitoneal injection of diclofenac for 4 weeks induced a significant elevation (*p* < 0.05) in serum ALT, AST, LDH, ALP, and GGT activities as well as serum total bilirubin level. The cotreatment of diclofenac-injected animals with naringin and/or hesperidin induced a decrease in these elevated levels. While the cotreatment of diclofenac-injected rats with naringin induced a significant decrease (*p* < 0.05) in the elevated serum AST, LDH, ALP, GGT, and total bilirubin levels, it produced a nonsignificant effect (*p* > 0.05) on the ALT activity. The cotreatment of diclofenac-injected animals with hesperidin induced a significant effect (*p* < 0.05) on the elevated ALT, AST, LDH, GGT, and total bilirubin levels while it failed to produce a significant effect in serum ALP activity (*p* < 0.05). The cotreatment with naringin and hesperidin in combination successfully caused a significant decrease (*p* < 0.05) in all elevated parameters related to liver function including ALT, AST, LDH, ALP, GGT, and total bilirubin levels. Moreover, the treatment with the combination of naringin and hesperidin appeared to be the most effective in improving the elevated AST, ALP, and GGT activities (Table 1). Serum albumin level, on the other hand, exhibited a nonsignificant change (*p* > 0.05) in all groups.

### 3.2. Effect on Liver Oxidative Stress and Antioxidant Defense System

The daily intraperitoneal injection of diclofenac for 4 weeks induced a significant elevation (*p* < 0.05) in liver LPO and a significant decrease (*p* < 0.05) in liver GSH content and GPx and SOD activities. The treatment of diclofenac-injected animals with naringin and/or hesperidin induced a significant decrease (*p* < 0.05) in liver LPO. Naringin and its combination with hesperidin were more potent than hesperidin alone in decreasing the elevated liver LPO product. While the treatment with naringin and/or hesperidin produced a significant increase (*p* < 0.05) in liver GPx and SOD activities, only naringin and hesperidin in combination induced a significant increase (*p* < 0.05) in liver GSH content (Table 2).

### 3.3. Effect on Liver p53 mRNA Expression

**Figure 2:**
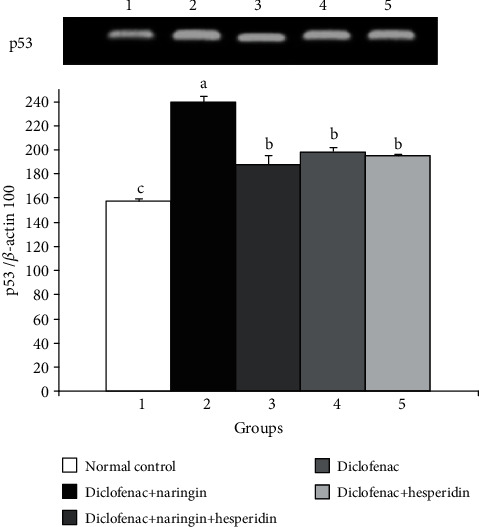
Effect of naringin, hesperidin, and their combination on p53 mRNA expression in the liver of diclofenac-administered rats. Means, which share different symbols, are significantly different at *p* < 0.05.

As indicated in Figure 2, the proapoptotic protein p53 mRNA expression significantly increased (*p* < 0.05) in the liver of diclofenac-injected rats. On the other hand, the treatment with naringin, hesperidin, and their combination produced a significant (*p* < 0.05) decrease in p53 mRNA expression in comparison with diclofenac-injected control. There is no significant difference between the three treatments when compared with each other.

### 3.4. Effect on Serum TNF-*α*, IL-17, and IL-4 Levels

In Table 3, the daily intraperitoneal injection of diclofenac for 4 weeks induced a significant increase (*p* < 0.05) in serum TNF-*α* and IL-17 levels and a significant decrease in serum IL-4 level. The cotreatment of diclofenac-injected rats with naringin and/or hesperidin induced a significant improvement (*p* < 0.05) of altered serum TNF-*α*, IL-17, and IL-4 levels. The treatment with the combination of both flavonoids was the most potent in increasing the lowered IL-4 level; the effect of the combination was more significantly potent (*p* < 0.05) as compared with the effect of either naringin or hesperidin.

### 3.5. Liver Histopathological Findings

**Figure 3:**
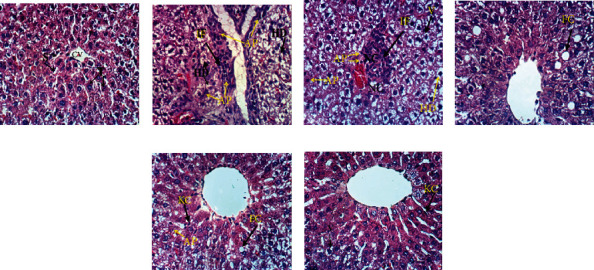
Photomicrographs of liver sections of the normal (a), diclofenac-injected group (b, c), and diclofenac-injected groups treated with naringin (d), hesperidin (e), and their combination (f). T: trabeculae; S: sinusoids; CV: central vein; IF: inflammatory cell infiltration; HD: hydropic degeneration; AP: apoptosis; NC: necrosis; FC: fatty changes; V: vacuolization; KC: Kupffer cells (H&E; ×400).

Figure 3 depicts the effect of naringin and/or hesperidin on liver histological changes of diclofenac-injected rats.  Figure 3(a) shows the liver with normal histological structure of hepatic lobule. On the other hand, the liver of diclofenac-injected rats exhibited marked deleterious histological changes like hydropic degeneration of hepatocytes, apoptotic cells, and portal infiltration with mononuclear cells (Figure 3(b)) in addition to cytoplasmic vacuolization of hepatocytes and focal hepatic necrosis associated with focal inflammatory cells infiltration (Figure 3(c)). These alterations were amended to some extent in the diclofenac-injected rats treated with naringin showing only fatty change of sporadic hepatocytes (Figure 3(d)). The treatment of diclofenac-injected rats with hesperidin produced an improvement of the liver histological changes as compared with the diclofenac-injected control but still exhibited fatty change of sporadic hepatocytes, slight activation of Kupffer cells, and presence of few apoptotic cells (Figure 3(e)). Similarly, the treatment of diclofenac-injected rats with a combination of naringin and hesperidin improved the liver histological changes as compared with diclofenac-injected control. However, the liver of diclofenac-injected rats with a combination of both flavonoids exhibited slight Kupffer cell proliferation and slight cytoplasmic vacuolization (Figure 3(f)). It seemed that the combinatory effect of naringin and hesperidin was the most potent in improving the liver histological architecture.

Histopathological change scores of all groups are recorded in Table 4. The liver section of normal control showed zero score for all histological lesions. The liver diclofenac-injected rats exhibited various grades of histological lesion scores ranging from grade III to grade 0. The administration of hesperidin and naringin to diclofenac-injected rats resulted in remarkable improvements in histological lesions including hydropic and vacuolar degeneration, inflammatory cells infiltration, necrosis, apoptosis, Kupffer cell proliferation, fatty changes and steatosis, congested blood vessels and sinusoids, and hyperplasia of epithelial lining of the bile duct; thereby, the liver exhibited lower grades of histopathological change scores in the diclofenac-injected groups treated with naringin and/or hesperidin in comparison with diclofenac-injected control rats. The combinatory effect of naringin and hesperidin was the most potent in decreasing the histopathological lesion scores.

### 3.6. Immunohistochemical Assay

**Figure 4:**
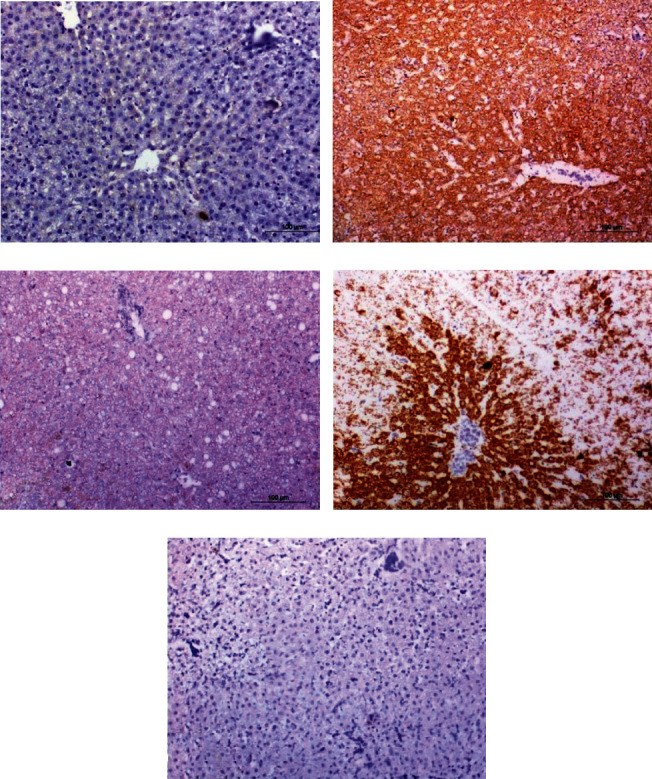
Immunohistochemical stained liver sections showing caspase-3 expression in the liver of the normal (a), diclofenac-injected group (b), and diclofenac-injected groups treated with naringin (c), hesperidin (d), and their combination (e) (×200).

**Figure 5:**
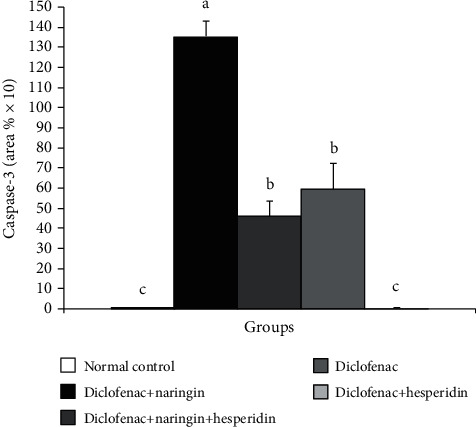
Immunohistochemical staining intensity of caspase-3 in the liver of the normal control, diclofenac control group, and diclofenac groups treated with naringin and/or hesperidin. Means, which share different symbols, are significantly different at *p* < 0.05.

The immunohistochemically stained liver sections depicted in Figure 4 and the results of image analysis are demonstrated in Figure 5.

The photomicrographs in Figure 4 and data in Figure 5 revealed that caspase-3 expression was significantly increased in diclofenac-injected rats. The treatment of diclofenac-injected rats with naringin, hesperidin, and their combination resulted in a significant decrease in the elevated caspase-3 expression in the liver. The combinatory effect of naringin and hesperidin was the most potent in decreasing the elevated caspase-3 content. The treatment with the combination normalized the liver caspase-3 expression.

## 4. Discussion

Diclofenac sodium is a well-known representative of drugs (NSAIDs) and it is widely used to control pain and inflammation of rheumatic and nonrheumatic origin [35]. It had been linked to serious side effects including gastric ulcers and liver, renal, and heart injuries [36–38]. Citrus fruit is one of the mostly consumed fruits worldwide, and numerous studies had revealed its remarkable health-promoting activities, such as antioxidant, anticancer, anti-inflammatory, and cardiovascular protection activities [39]. Such activities largely depend on the diverse chemical constituents of citrus fruits, including vitamins, minerals, terpenoids, and flavonoids which had attracted growing interest due to their distinct beneficial effects on human health. The daily ingested fruits and vegetables are rich sources of both nutrients such as carbohydrate, vitamins, and minerals, and nonnutritive constituents, particularly polyphenols including flavonoids and phenolic acids [40]. Flavonoids especially flavanones, which contain hesperidin and naringin, are well known for their antioxidant properties and as health-promoting molecules with multifunctional biological activities; they had been shown to attenuate inflammation, to quench active oxygen species [41] and to prevent liver, kidney, and heart toxicities as well as several forms of cancer [42–44].

The present study was conducted to evaluate the preventive effects of the naringin, hesperidin, and their combination against diclofenac-induced liver injury in rats and to elucidate the mode of actions by assessing the effects on oxidative stress, antioxidant defense system, inflammation, and apoptosis.

**Figure 6:**
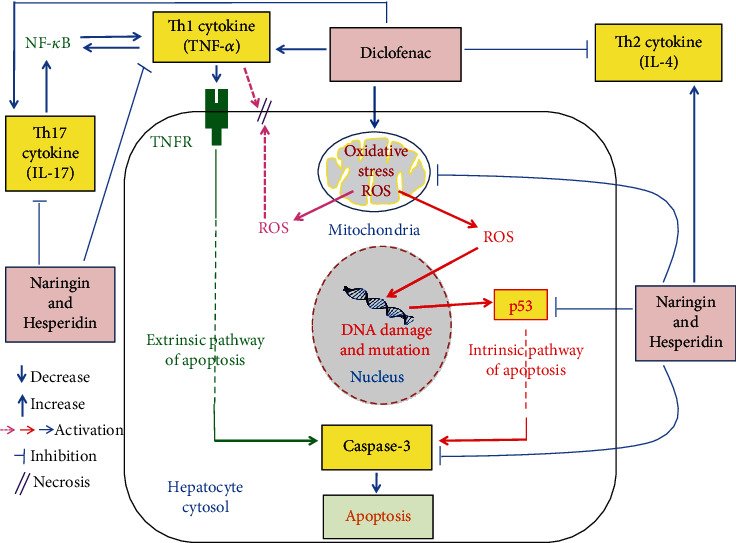
Schematic diagram showing the effects of naringin and hesperidin to suppress oxidative stress, inflammation, and apoptosis in diclofenac-injected rats.

The present study showed that diclofenac administration profoundly produced a significant increase in serum enzyme (ALT, AST, LDH, ALP, and GGT) activities, bilirubin level, and proinflammatory cytokine (TNF-*α* and IL-17) levels as well as liver LPO, caspase-3, and p53 mRNA expression but it induced a significant decrease in serum anti-inflammatory cytokine (IL-4) level and liver GSH content as well as SOD and GPx activities. These alterations reflect hepatocyte damage and necrosis, biliary liver dysfunction, increase in oxidative stress, suppression of antioxidant defense system, and augmentation of inflammation and apoptosis. The present study is in agreement with many previous publications [45–51]. The present biochemical and pathophysiological alterations in diclofenac-injected rats are associated with liver histological alterations including hydropic and vacuolar degeneration of hepatocytes, inflammatory cells' infiltration, focal hepatic necrosis, apoptosis, congestion of blood vessels and sinusoids, fatty changes, Kupffer cells' proliferation, and hyperplasia of the epithelial lining the bile canaliculi. The histological scores of these lesions were much lower in diclofenac-injected rats treated with naringin and/or hesperidin than those in diclofenac-injected control; the effect of combination was the most potent in decreasing the lesions' scores. The previous histological lesions in diclofenac-injected rats may be attributed to the increase in the oxidative stress and excessive release of free radicals and ROS. This exacerbated production of ROS administration activates apoptosis through intrinsic pathway, aggravates cell necrosis through peroxidation of membrane lipids, and stimulates DNA breakages and mutations by oxidative damage [43, 52] (Figure 6). In addition to its necrotic effects, the elevation of TNF-*α* may augment hepatocyte apoptosis through linking to TNF receptor (TNFR) and death receptors leading to activation of extrinsic pathway of apoptosis [43, 52, 53] (Figure 6). In turn, the improvement effects of naringin and hesperidin on liver histological lesions as well as ameliorations of serum biochemical parameters may be due to the suppressive effects of these two flavonoids on oxidative stress and enhancement of the antioxidant defense system in addition to the suppressive effects on inflammatory mediators such as TNF-*α* and IL-17 (Figure 6). In agreement with our results, GökÇimen et al. [54], Tan et al. [55], and El-Kordy and Makhlouf [56] revealed hepatocyte necrosis and interstitial and periportal inflammation, which indicate acute hepatitis in diclofenac-administered animals. Also, Alqasoumi [49] reported that the diclofenac-treated rats revealed early bridging necrosis and lymphocytic infiltrate.

In the current study, the serum albumin levels were not significantly altered in all groups. The serum albumin reflects the synthetic capacity of liver cells as albumin is formed by liver cells (hepatocytes). The nonsignificant change in serum albumin level in the diclofenac group in spite of the significant increase in serum enzymes, mostly or partially leaked from the liver to plasma, led us to suggest that the stressed and survived hepatocytes may have increased capability to synthesize more albumin as a compensatory mechanism.

In the present study, the inflammatory cell infiltration in the liver of diclofenac-injected rats was associated with an elevation in serum levels of inflammatory cytokines (TNF-*α* and IL-17) and a decrease in serum level of anti-inflammatory cytokine, IL-4. These findings are in agreement with many previous publications. Hussien et al. [50] reported that oral administration of diclofenac sodium led to a significant increase in serum TNF-*α* level. Deng et al. [57], Schmidt-Weber et al. [58], and Ahmed et al. [59] showed that overproduction of TNF-*α* and IL-17 had been implicated in the pathogenesis of several inflammatory conditions by inducing and maintaining inflammation. Moreover, IL-17 stimulates inflammatory responses *via* NF-*κ*B, which plays the important role as the key regulator of transcriptional responses to TNF-*α* [60, 61]. On the other hand, IL-4 is the complicated cytokine whose role varies between anti- and proinflammation in autoimmunity. IL-4 had an immunoregulatory role in immune-mediated drug-induced liver injury; this represents a unique condition where hepatitis can be initiated by drug haptens or self-proteins [62]. In the current study, the diclofenac has a suppressive effect on IL-4 production in Wistar rats.

The elevation in the TNF-*α* level in diclofenac-injected rats may activate extrinsic apoptotic pathway through TNF receptor (TNFR) and death receptor. On the other hand, the increased mRNA expression of p53 in association with the increase in oxidative stress may reflect the stimulation of intrinsic pathway. Furthermore, caspase-3, which serves as a convergence point for both intrinsic and extrinsic pathways, is significantly elevated in diclofenac-injected rats. Thus, it can be suggested that diclofenac may activate apoptosis in Wistar rats *via* stimulation of both apoptotic pathways (Figure 6).

The oral administration of naringin and/or hesperidin to diclofenac-injected rats produced a marked decrease in the elevated serum ALT, AST, LDH, ALP, and GGT activities and serum total bilirubin level reflecting improvement of liver function and integrity; the combinatory effect seemed to be the most potent on AST, ALP, and GGT activities. These improvements in biochemical parameters related to liver function were associated with alleviations in liver histological architecture; the treatment with the combination was the most effective. Such decrease in the elevated serum ALT, AST, LDH, ALP, and GGT activities and serum total bilirubin level as well as alleviated liver histological architecture and integrity were accompanied with a decrease in the elevated liver LPO, serum Th1 cytokine (TNF-*α*), serum Th17 cytokine (IL-17), and liver proapoptotic mediators (p53 and caspase-3) expression. On the other hand, serum Th2 cytokine (IL-4) level, liver GSH content, and antioxidant enzyme (GPx and SOD) activities were enhanced by treatment of diclofenac-injected rats with naringin and/or hesperidin. These results go parallel with Ahmed et al. [43] who found that the administration of naringin to acetaminophen-supplemented rats improved the deteriorated changes in serum ALT, AST, LDH, ALP, GGT, TNF-*α*, and IL-4 levels as well as liver LPO and antioxidant defense system. With regard to apoptosis, Ahmed et al. [43] reported decrease in apoptotic proteins p53 and caspase-3 as result of treatment of acetaminophen-injected rats with naringin and naringenin. The present results are also in concurrence with Omar et al. [63] who found that hesperidin significantly reduced cisplatin-induced elevations in serum ALT and AST activities as well as liver LPO, NO content, and NF-*κ*B. The NF-*κ*B has the important role as the key regulator of transcriptional responses to TNF-*α* expression (Figure 6). In the same regard, Çetin et al. [64] stated that hesperidin caused an increase in liver GSH, catalase, and SOD levels and induced a decrease in LPO in the liver of CCl4-injected rats. Thus, based on the results of the present study and of past publications, it can be suggested that the ameliorative effects of naringin and hesperidin, singly or in combination, on liver function and structural integrity may be attributed to their suppressive effects on the inflammation, oxidative stress, and apoptosis and to their stimulatory effects on anti-inflammatory effects on antioxidant defense system (Figure 6). This suggestion was supported by previous publications which stated the antioxidant and anti-inflammatory effects of naringin and hesperidin in different diseased conditions and drug toxicities [65–75].

Naringin and hesperidin may decrease apoptosis in diclofenac-injected rats through extrinsic pathway and/or intrinsic pathway. In the present study, naringin and hesperidin treatment of diclofenac-injected rats resulted in a decrease in TNF-*α* which in turn activates extrinsic apoptotic pathway through TNFR and death receptor. Naringin and hesperidin treatment of diclofenac-injected rats also caused a decrease in mRNA expression of p53 which is mediator in intrinsic pathway. In addition, naringin and hesperidin induced a decrease in liver caspase-3, which is a convergence point for both intrinsic and extrinsic pathways (Figure 6).

Overall, it can be concluded that multiple long-term diclofenac administration induces liver toxicity. Administration of hesperidin, naringin, and their combination, which was the most potent, potentially counteracts diclofenac-induced liver injury and toxicity *via* enhancement of antioxidant defense system and anti-inflammatory effect as well as suppression of oxidative stress and apoptosis. However, further clinical investigations are required to assess the efficacy and safety of hesperidin and naringin in human beings.

## Figures and Tables

**Table 1:** Effect of naringin, hesperidin, and their combination on serum biochemical parameters related to liver function in diclofenac-administered rats.

Parameter	Normal control	Diclofenac	Diclofenac+naringin	Diclofenac+hesperidin	Diclofenac+naringin+hesperidin
ALT (U/L)	48.0 ± 1.34^c^	62.00 ± 0.89^a^	58.83 ± 0.94^ab^	51.00 ± 0.63^c^	55.50 ± 2.4^b^
AST (U/L)	138.20 ± 5.11^b^	244.00 ± 30.44^a^	163.83 ± 16.31^b^	141.00 ± 6.76^b^	136.67 ± 8.46^b^
LDH (U/L)	366.00 ± 51.42^c^	786.00 ± 18.33^a^	615.66 ± 65.19^b^	580.66 ± 14.53^b^	669.00 ± 1.79^b^
ALP (U/L)	193.66 ± 18.92^c^	329.33 ± 33.80^a^	259.83 ± 9.10^b^	270.17 ± 21.05^ab^	226.33 ± 10.83^bc^
GGT (U/L)	5.30 ± 1.10^b^	17.0 ± 0.80^a^	5.00 ± 1.00^b^	7.00 ± 1.00^b^	4.50 ± 0.80^b^
Albumin (g/dL)	3.0 ± 0.04^a^	3.03 ± 0.02^a^	2.95 ± 0.09^a^	3.02 ± 0.05^a^	2.90 ± 0.04^a^
Bilirubin (mg/dL)	0.30 ± 0.04^b^	0.50 ± 0.04^a^	0.33 ± 0.02^b^	0.30 ± 0.04^b^	0.35 ± 0.05^b^

Data are expressed as the mean ± SE. Number of animals in each group is six. Means, which share different superscript symbols in the same row, are significantly different at *p* < 0.05.

**Table 2:** Effect of naringin, hesperidin, and their combination on liver LPO and antioxidant defense markers in diclofenac-administered rats.

Parameter	Normal control	Diclofenac	Diclofenac+naringin	Diclofenac+hesperidin	Diclofenac+naringin+hesperidin
LPO (MDA nmol/g tissue)	181.75 ± 5.92^c^	386.90 ± 14.08^a^	174.63 ± 14.83^c^	265.03 ± 29.66^b^	194.35 ± 19.22^c^
GSH (nmol/100 mg tissue)	30.50 ± 2.47^a^	19.73 ± 1.0^c^	20.80 ± 0.79^bc^	22.68 ± 0.92^bc^	23.73 ± 0.77^b^
GPx (mU/100 mg tissue)	175.00 ± 4.08^a^	46.33 ± 8.34^c^	151.17 ± 13.19^ab^	140.00 ± 11.90^b^	135.00 ± 11.47^b^
SOD (U/g tissue)	18.43 ± 0.37^a^	12.28 ± 0.31^c^	15.98 ± 0.75^b^	15.65 ± 0.54^b^	16.56 ± 0.68^b^

Data are expressed as the mean ± SE. Number of animals in each group is six. Means, which share different superscript symbols in the same row, are significantly different at *p* < 0.05.

**Table 3:** The effect of naringin, hesperidin, and their combination on serum TNF-*α*, IL-17, and IL-4 levels in diclofenac-administered rats.

Serum parameter	Normal control	Diclofenac	Diclofenac+naringin	Diclofenac+hesperidin	Diclofenac+naringin+hesperidin
TNF-*α* (pg/mL)	33.07 ± 0.51^c^	154.12 ± 11.63^a^	102.95 ± 3.44^b^	88.08 ± 2.37^b^	91.50 ± 4.67^b^
IL-17 (pg/mL)	40.05 ± 3.16^c^	151.48 ± 3.60^a^	83.30 ± 1.27^b^	77.60 ± 4.26^b^	77.60 ± 3.99^b^
IL-4 (pg/mL)	130.00 ± 3.77^a^	70.37 ± 3.12^d^	100.53 ± 0.60^c^	106.43 ± 2.65^c^	116.88 ± 2.16^b^

Data are expressed as the mean ± SE. Number of animals in each group is six. Means, which share different superscript symbols in the same row, are significantly different at *p* < 0.05.

**Table 4:** Histopathological scores of liver lesions in the normal control, diclofenac-injected group, and diclofenac-injected groups treated with naringin, hesperidin, and their combination.

Histopathological changes	Score	Normal control	Diclofenac	Diclofenac+naringin	Diclofenac+hesperidin	Diclofenac+naringin+hesperidin
Hydropic and vacuolar degeneration	0	6 (100%)	1 (16.7%)	3 (50.0%)	2 (33.3%)	4 (66.7%)
	I	—	1 (16.7%)	2 (33.3%)	2 (33.3%)	2 (33.3%)
	II	—	2 (33.3%)	1 (16.7%)	2 (33.3%)	—
	III	—	2 (33.3%)	—	—	—
Inflammation (inflammatory cell infiltration)	0	6 (100%)	—	3 (50.0%)	3 (50.0%)	5 (83.3%)
	I	—	1 (16.7%)	1 (16.7%)	2 (33.3%)	1 (16.7%)
	II	—	2 (33.3%)	2 (33.3%)	1 (16.7%)	—
	III	—	3 (50.0%)	—	—	—
Necrosis	0	6 (100%)	—	1 (16.7%)	2 (33.3%)	4 (66.7%)
	I	—	2 (33.3%)	2 (33.3%)	3 (50.0%)	2 (33.3%)
	II	—	2 (33.3%)	3 (50.0%)	1 (16.7%)	—
	III	—	2 (33.3%)	—	—	—
Apoptosis	0	6 (100%)	—	3 (50.0%)	3 (50.0%)	4 (66.7%)
	I	—	1 (16.7%)	3 (50.0%)	2 (33.3%)	1 (16.7%)
	II	—	2 (33.3%)	—	1 (16.7%)-	1 (16.7%)
	III	—	3 (50.0%)	—	—	—
Kupffer cell proliferation	0	6 (100%)	—	3 (50.0%)	3 (50.0%)	4 (66.7%)
	I	—	1 (16.7%)	1 (16.7%)	2 (33.3%)	1 (16.7%)
	II	—	3 (50.0%)	2 (33.3%)	1 (16.7%)	1 (16.7%)
	III	—	2 (33.3%)	—	—	—
Fatty changes and steatosis	0	6 (100%)	4 (66.7%)	4 (66.7%)	4 (66.7%)	6 (100%)
	I	—	1 (16.7%)	1 (16.7%)	—	—
	II	—	1 (16.7%)	1 (16.7%)	2 (33.3%)	—
	III	—	—	—	—	—
Congested blood vessels and sinusoids	0	6 (100%)	—	3 (50.0%)	4 (66.7%)	4 (66.7%)
	I	—	2 (33.3%)	—	1 (16.7%)	2 (33.3%)
	II	—	3 (50.0%)	2 (33.3%)	1 (16.7%)	—
	III	—	1 (16.7%)	1 (16.7%)	—	—
Hyperplasia of epithelial lining of the bile duct	0	6 (100%)	4 (66.7%)	5 (83.3%)	6 (100%)	6 (100%)
	I	—	—	—	—	—
	II	—	—	1 (16.7%)	—	—
	III	—	2 (33.3%)	—	—	—

0: absence of lesion; I: mild; II: moderate; III: severe. Number of animals in each group is 6. Numbers 1, 2, 3, 4, 5, and 6 represent the number of animals for each grade. The % in parentheses is the percent of animals in each grade.

## Data Availability

The data used to support the findings of this study are available from the corresponding author upon reasonable request.
